# HERV-K activation is strictly required to sustain CD133+ melanoma cells with stemness features

**DOI:** 10.1186/s13046-016-0485-x

**Published:** 2017-01-26

**Authors:** Ayele Argaw-Denboba, Emanuela Balestrieri, Annalucia Serafino, Chiara Cipriani, Ilaria Bucci, Roberta Sorrentino, Ilaria Sciamanna, Alessandra Gambacurta, Paola Sinibaldi-Vallebona, Claudia Matteucci

**Affiliations:** 1 Department of Experimental Medicine and Surgery, University of Rome “Tor Vergata”, Via Montpellier 1, 00133 Rome, Italy; 2 Institute of Translational Pharmacology, National Research Council, Via Fosso del Cavaliere 100, 00133 Rome, Italy; 3 S.B.G.S.A. Istituto Superiore di Sanità (Italian National Institute of Health), Viale Regina Elena 299, 00161 Rome, Italy

**Keywords:** Cancer stem cell, CD133, HERV-K, Melanoma, Microenvironment, Retroelements

## Abstract

**Background:**

Melanoma is a heterogeneous tumor in which phenotype-switching and CD133 marker have been associated with metastasis promotion and chemotherapy resistance. CD133 positive (CD133+) subpopulation has also been suggested as putative cancer stem cell (CSC) of melanoma tumor. Human endogenous retrovirus type K (HERV-K) has been described to be aberrantly activated during melanoma progression and implicated in the etiopathogenesis of disease. Earlier, we reported that stress-induced HERV-K activation promotes cell malignant transformation and reduces the immunogenicity of melanoma cells. Herein, we investigated the correlation between HERV-K and the CD133+ melanoma cells during microenvironmental modifications.

**Methods:**

TVM-A12 cell line, isolated in our laboratory from a primary human melanoma lesion, and other commercial melanoma cell lines (G-361, WM-115, WM-266-4 and A375) were grown and maintained in the standard and stem cell media. RNA interference, Real-time PCR, flow cytometry analysis, self-renewal and migration/invasion assays were performed to characterize cell behavior and HERV-K expression.

**Results:**

Melanoma cells, exposed to stem cell media, undergo phenotype-switching and expansion of CD133+ melanoma cells, concomitantly promoted by HERV-K activation. Notably, the sorted CD133+ subpopulation showed stemness features, characterized by higher self-renewal ability, embryonic genes expression, migration and invasion capacities compared to the parental cell line. RNA interference-mediated downregulation experiments showed that HERV-K has a decisive role to expand and maintain the CD133+ melanoma subpopulation during microenvironmental modifications. Similarly, non nucleoside reverse transcriptase inhibitors (NNRTIs) efavirenz and nevirapine were effective to restrain the activation of HERV-K in melanoma cells, to antagonize CD133+ subpopulation expansion and to induce selective high level apoptosis in CD133+ cells.

**Conclusions:**

HERV-K activation promotes melanoma cells phenotype-switching and is strictly required to expand and maintain the CD133+ melanoma cells with stemness features in response to microenvironmental modifications.

## Background

The origin of intratumoral heterogeneity in melanoma is not yet clearly defined. However, studies are revealing that many cancers, including melanoma, are hierarchically organized and driven by a particular subpopulation of cancer cells that have stem cells properties, known as cancer stem cells (CSCs) [1]. CSCs have been defined by their high self-renewal capacity and tumorigenic potential leading to tumor initiation, metastasis, therapeutic resistance and tumor recurrence. Single or multiple intra- or extra-cellular markers have been used to identify CSCs in different types of tumors [1, 2]. In melanoma, different markers have been employed to characterize the putative melanoma CSC and, particularly, CD133, CD271, CD146, ALDH, ABCB5, ABCG2, Nestin and CD117 were considered [3, 4]. Although the specificity of these markers are still under debate, the stem cell marker CD133 has been widely used to characterize and isolate the putative melanoma CSC in both in vitro and in vivo studies [4, 5]. In fact, CD133 was shown to be expressed in human melanoma biopsies but hardly detected in normal skin sections [3, 5]. Furthermore, it was revealed that CD133^+^ melanoma cells have an enhanced capability to initiate primary tumors and metastasis in NOD/SCID mice, while showing higher self-renewing and migration capacity and differentiation potential into mesenchymal lineages in in vitro studies [4, 6, 7]. Moreover, the CD133 marker has been associated with promotion of vasculogenic mimicry and angiogenesis, and was found to inhibit tumor cell apoptosis by interacting directly with the vascular endothelial growth factor (VEGF) [8, 9]. Intriguingly, recent studies revealed that CD133+ melanoma cells are highly resistant to chemotherapy, indicating that they are also responsible for tumor recurrence [2, 10]. Likewise, monoclonal antibodies directed against CD133 protein induced a specific dose-dependent cytotoxic effect in metastatic melanoma cells, suggesting CD133 as a potential target for immunotherapy [11]. Recent evidence also indicated knocking down CD133 in NRASQ^61R^/BRAF^WT^ mutant melanoma renders cells more sensitive to clinically employed-MEK/BRAF inhibitors [12].

Beyond the notion that melanoma growth and progression is sustained by stem cell-like cancer cells, it is a well-evidenced fact that melanoma is driven by a diverse group of genetic and epigenetic lesions and signaling pathways that are imposed by change in microenvironmental conditions [13, 14]. Nevertheless, in melanoma, as well in other type of cancers, protein coding gene mutations are no longer considered as sole drivers of the disease. For these reasons, a growing interest is now focused on the role of mobile genetic elements in tumor initiation and progression. These mobile genetic elements, primarily retroelements, comprise nearly half of the human genome. Retroelements, unless tightly regulated, may cause insertional mutagenesis or transcriptional dysregulation that potentially induces cellular transformation and tumorigenesis [15, 16].

Human endogenous retroviruses (HERVs) belong to retroelements and are remnants of ancient retrovirus infections that actually constitute about 9% of the human genome [16]. Most HERVs are defective but there are few active HERVs that are dynamically expressed and epigenetically regulated in a stage-dependent manner during early embryonic development. In somatic tissues, some members of HERV family remain transcriptionally active and display tissue-specific expression [16–18]. So far, HERVs have been implicated in both biological and pathological processes such as in cancer, diabetes and neuropsychiatric diseases, but their specific pathophysiological roles are still poorly defined [19–22]. Among HERV families, HERV-K is the youngest and most active family that maintains most of the open reading frames (ORFs) analogous to many active retroviruses [15, 22]. HERV-K viral particles have been identified and characterized at tissue, serum and cell lines level in association with different types of tumors, including ovarian, breast and prostate cancer, teratocarcinoma, lymphomas, leukemia, sarcomas and in more recent years with melanoma [23, 24]. The oncogenic mechanisms of HERV-K may depend from the expression of gene products potentially carcinogenic or immune escape causative, or from the regulatory role of their long terminal repeats (LTRs) sequences for the nearby (proto-) oncogenes or growth factors [15, 16, 22].

Several studies have shown that HERV-K is aberrantly activated during melanoma progression, contributes to cell malignant transformation and promotes immune escape during metastasis formation [23–28]. HERV-K *Rec* and *Np9* accessory proteins, described as putative oncogenes, have been associated with carcinogenesis by interacting with proteins involved in cellular transformation [29–31]. Likewise, HERV-K env protein may increase the risk of melanoma cancer by disrupting normal intracellular redox potential resulting in rise of toxic free radicals [32]. Furthermore, HERV-K proteins have been shown to suppress the host immune system [33, 34]. Recent studies also suggested the env protein of HERV-K might be a key mediator, at least partly, in the constitutive activation of the RAS-RAF-MEK pathway, which is aberrantly activated in over 80% of all cutaneous melanomas [34–36].

Previously we demonstrated for the first time that HERV-K activation induced melanoma cell malignant transformation and reduced the immunogenicity of melanoma cells that favors tumor immune escape [26]. Herein, we show that melanoma cells exposed to stem cell media were compelled to undergo phenotype-switching towards greater malignancy and increment of stem cell related features concomitant to HERV-K activation. These phenomena are reversible and promoted by HERV-K activation. Moreover, this study revealed that HERV-K activation is strictly required to sustain CD133+ melanoma cells with stemness features during microenvironmental modifications.

## Methods

### Cell lines and culture conditions

In this study the human melanoma primary tumor derived WM-115 cell line, and its metastasis derived counterpart WM-266-4, the malignant human melanoma cell lines G-361, A375 (all from ATCC, Manassas, VA, USA) and the human melanoma TVM-A12 cell line, stabilized in our laboratory, were used [37]. TVM-A12-CD133^+^ cells were sorted and isolated from TVM-A12 cell line. All cell lines were cultured as adherent cells in RPMI-1640 medium supplemented with 10% (*v/v*) heat-inactivated fetal bovine serum (FBS), L-glutamine (2 mM), Penicillin-Streptomycin (100 mg/ml) at 37 °C in a humidified 5% CO_2_ atmosphere and serially passaged twice weekly after detachment with 0.05% trypsin and 0.02% EDTA solution in PBS (all reagents from Sigma-Aldrich, St Louis, MO, USA). To assess cellular plasticity and malignant phenotype switching under microenvironmental modifications, cells were cultured in the serum-free stem cells medium X-VIVO™ 15 (Lonza, Verviers, Belgium) supplemented with Penicillin-Streptomycin (100 IU/ml).

### Flow cytometry analysis

For cytofluorimetric analysis adherent cells were trypsinized, then washed twice in PBS, incubated with 5 μl of fluorochrome-conjugated antibodies for 30 min at 4 °C (5 × 10^5^cells/FACS tube), fixed with 1% formaldehyde solutions for 5 min at 4 °C, centrifuged for 5 min at 1600 rpm and washed once with 1 ml of PBS for 5 min at 1600 rpm. CD133/2 (293C3) (Miltenyi Biotec, Bergisch Gladbach, Germany), HLA-I, NGF-R, ICAM-1, CD20, CXCR4, CD10, c-Kit antibodies (all from BD Biosciences, Franklin Lakes, NJ, USA) conjugated with different fluorescent dyes were used; the unconjugated antibodies Nestin (Novus Biologicals, Minneapolis, USA) and Melan-A/MART-1 (Santa Cruz Biotechnology, Dallas, TX, USA) were used in combination of the goat-anti-mouse IgG-FITC (BD Biosciences) as secondary antibody and used after cell permeabilization. For apoptosis analysis cells were trypsinized, washed in PBS, fixed with 70% ethanol for 45 min at 4 °C, washed in PBS and stained with propidium iodide (50 μg/ml diluted in PBS) and RNAase (250 μg/ml), then stored for at least 3 h at 4 °C before analysis. Flow cytometer analysis was performed by BD FACScan™ System using CellQuest Pro software on a minimum of 5000 events for each sample.

### Microscopic and side population analyses

Morphological analysis was carried out by phase-contrast microscopy, using the Motic AE31 Trinocular inverted microscope (Motic Asia, Hong Kong).

The differential ability of melanoma cells to efflux the Hoechst dye [38] was evaluated by Hoechst 33342 extrusion test. In detail, TVM-A12 cells were grown in X-VIVO medium to induce cellular aggregates. The obtained cellular aggregates were grown on coverslips at a concentration of 10^6^ cells/ml in X-VIVO medium in presence of 2% FBS, stained at 37 °C for 90 min with 4 μg/ml Hoechst 33342 (Sigma-Aldrich), and analyzed without fixing under the ZEISS Axioplan fluorescence microscope (Oberkochen, Germania) equipped with a digital camera.

For side population (SP) analysis by flow cytometry, we adopted the original SP protocol [39] and performed the analysis as described before [40]. Briefly, TVM-A12 cells cultured in RPMI 10% FBS medium or in X-VIVO for 72 h were adjusted to 10^6^ cells/ml concentration and incubated with 5 μg/ml Hoechst 33342 nucleic acid stain for 90 min at 37 °C. To reduces efflux of the Hoechst dye and confirm the SP phenotype, Verapamil (50 μM; Sigma-Aldrich) was added 30 min before Hoechst stain in control samples. For further SP characterization, after Hoechst staining, cells were maintained in ice and immunostained with CD133 or isotype antibodies (Miltenyi Biotec). Finally, cells were resuspended in ice-cold staining buffer (PBS + 2%FBS) for subsequent analysis by flow cytometry using a FACSCanto II (BD Biosciences).

### Flow cytometry sorting

For fluorescence-activated cell sorting, TVM-A12 cells, cultivated in X-VIVO, were enzymatically dissociated and stained by incubation with PE-conjugated monoclonal antibody against CD133 and/or PE- isotype mouse IgG2b at 4 °C for 1 h. After staining, samples were washed twice with PBS, resuspended at 2 × 10^6^ cells/ml in PBS and filtered (50 μm, Partech). Live cell sorting experiments were performed using BD FACSAria II (Becton Dickinson Immunocytometry Systems) with 130 μm nozzle and sort gates were defined on a dot plot of CD133 (PE). PE fluorescence of CD133 was determined by a 488 nm excitation line and detected by 585/42 nm filters. Sorted cells were collected in PBS medium. The samples were analyzed using the FACSDiva software (Becton Dickinson).

### Sphere formation assay

A total of 500 single viable cells (TVM-A12 and TVM-A12-CD133+) were seeded into 48-well tissue culture plates coated with 0.5 mg/ml of poly-2-hydroxyethyl methacrylate (Poly-HEMA, Sigma) in a 500 μl of serum-free DMEM/F12 (1:1) (Sigma) basal medium supplemented with L-glutamine (2 mM), Penicillin-Streptomycin (100 mg/ml), 20 ng/ml human epidermal growth factor (EGF) 20 ng/ml, human fibroblast growth factor-2 (FGF-2) (ProSpec, Rehovot, Israel), and 1:50 B-27 supplement (Gibco_,_ Life Technologies, Carlsbad, CA, USA) and cultured at 37 °C in a humidified 5% CO_2_ for 10 days to form melanospheres. For serial passage, these melanospheres were counted using a manually prepared “quadrant grid” under microscopic observation, harvested and centrifuged at 1000 rpm for 5 min, trypsinized to dissociate in to single cell, counted and viable cells reseeded in the Poly-HEMA coated 48-well plates for subsequent passages.

### Migration and invasion assays

For migration assay, cells were maintained in serum-free RPMI-1640 medium for 18 h, harvested and resuspended in the same medium, and seeded into Bio-Coat cell migration chambers with 8 μm membrane pore sizes (BD Biosciences) at 1 × 10^5^ cells in 250 μl per chamber. The chambers were then inserted into the wells of a 24-well plate containing 750 μl of RPMI-1640 medium with 20% FBS alone or 20% FBS with 40 ng/ml human hepatocytes growth factor (HGF) (ImmunoTools, Friesoythe, Germany) and incubated at 37 °C with 5% CO_2_ for 48 h. After this period, the cells remaining on the upper surface of the membrane were removed with cotton swab and the cells adhered to the lower surface were fixed with 70% ethanol for 15 min, stained with Giemsa (Sigma) for further 15 min, then washed twice with water. After drying, the membrane was removed with surgical blade and mounted on glass slides. Pictures were taken with Olympus BX50 microscope from five random microscopic fields at 200x magnification, and cells counted with image analysis software (ImageJ, NIH). For invasion assay, the same procedure was followed except for the coating of the migration chambers with 50 μl (0.3 mg/ml) of Matrigel matrix (BD Biosciences) before of the cells seeding.

### RNA extraction and real-time PCR

Total cellular RNA was extracted using NucleoSpin RNA II kit (Machery-Nagel GmbH & Co. KG, Düren, Germany) following the manufacturer’s instructions. Reverse transcription was performed using the ImProm-II™ Reverse Transcription System Kit (Promega, Madison, USA) following the manufacturer’s instructions. The Real-time PCR gene-specific primers for HERV-K *env*, CD133, and the housekeeping gene beta-glucuronidase (GUSB) were purchased from Invitrogen Thermo Fisher Scientific (Waltham MA, USA). The PCR primers for each specific genes were as follows: env forward, 5′-GCCATCCACCAAGAAAGCA-3′; env reverse, 5′-AACTGCGTCAGCTCTTTAGTTGT-3′ (AF164614); CD133 forward, 5′-TTTCAAGGACTTGCGAACTCTCTT-3′; CD133 reverse, 5′-GAACAGGGATGATGTTGGGTCTCA-3′ (NM_001145848.1); Oct4 forward, 5′-TATGCAAAGCAGAAACCCTCGTGC-3′; Oct4 reverse, 5′-TTCGGGCACTGCAGGAACAAATTC-3′ (NM_002701); Nanog forward, 5′-TCCAGCAGATGCAAGAACTCTCCA-3′; Nanog reverse, 5′-CACACCATTGCTATTCTTCGGCCA-3′ (NM_024865); GUSB forward, 5′-CAGTTCCCTCCAGCTTCAATG-3′; GUSB reverse, 5′-ACCCAGCCGACAAAATGC-3′ (NM_000181). Real-time PCR was performed in CFX96 Real-time System using SsoAdvanced™ Universal SYBR Green Supermix (Bio-Rad Laboratories, Hercules, CA USA) amplification detection method. Samples were analyzed in duplicate and for each experiment no template controls (NTC) and GUSB were used as internal control and to determine the amplification efficiency, respectively. The threshold cycle (Ct) comparative method was used to analyze the relative changes in gene expression of each sample compared with the reference sample, calculating as follows:2^−[ΔCt(sample) − ΔCt(calibrator)]^ = 2^−ΔΔCt^, where ΔCt (sample) = [Ct (HERV-Kenv) – Ct (GUSB)], and ΔCt (calibrator) was the mean of ΔCt of TVM-A12 cells maintained in RPMI 10%FBS.

### RNA interference

HERV-K (AF164614) expression was down-regulated by infecting TVM-A12 cells with a retroviral vector as previously described [26, 27]. Briefly, Phoenix packaging cells were transfected with 9 μg of construct DNA using lipofectamine (Gibco-BRL, 18324–012). 48 h later, cell culture media were collected and filtered through 0.45 μm Millipore filter and used to infect TVM-A12 cells in the presence of 4 μg/ml polybrene (Sigma) for 12 h, after which the medium was changed. Infected cells were selected on 0.5 μg/ml puromycin (Sigma) for 6 days. After selection, the cells were grown in RPMI-1640 medium supplemented with 10% FBS and maintained under antibiotic selection (0.2 μg/ml).

### Reverse transcriptase inhibitors treatments

TVM-A12 and TVM-A12-CD133 cells were cultured for 24 h in RPMI-1640 medium with 10% FBS at 37 °C and 5% CO_2_ to ensure cells form a monolayer before starting treatment. Then, cells were cultivated in RPMI-1640 with 10% FBS, or in serum-free stem cell media (X-VIVO™ 15) to induce the grape-like cellular aggregates formation. The non-nucleoside reverse transcriptase (RT) inhibitors (NNRTIs) nevirapine (NVP) or efavirenz (EFV) were added to the cultures at 350 μM and 15 μM respectively. Dimethyl sulfoxide (DMSO) was used as diluent for the drugs and was referred as vehicle in control condition. After 72 h of treatment cells were detached (trypsin used only for adherent cells) and back cultured with the same fresh media for the next 24 h and retreated with NVP or EFV at half concentration of initial used. Finally cells from each condition were collected and processed for Real-time PCR and flow cytometry analysis.

### Statistical analysis

Data analysis was performed using the SPSS statistical software system (version 17). Comparison of means was carried out using Bonferroni post-hoc multiple comparison Anova test. Statistical probabilities were expressed as *p* ≤0.050 (*) or *p* < 0.001 (**).

## Results

### TVM-A12 human melanoma cells led to phenotype switching and expansion of the CD133^+^ melanoma subpopulation when cultured in stem cell medium

**Fig. 1:**
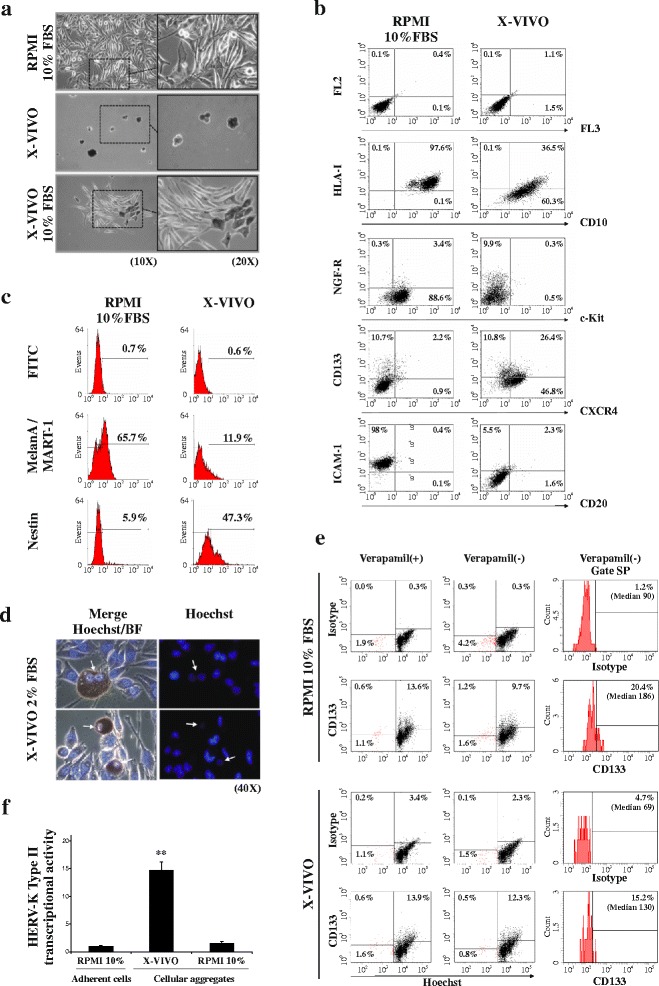
TVM-A12 cells exhibit adaptive plasticity, phenotype switching and concomitant up-regulation of HERV-K expression upon microenvironmental modifications. **a** Phase contrast microscopy of TVM-A12 grown in RPMI-1640 complete medium, in stem cell medium (X-VIVO) or upon the addition of increasing percentage of serum (up to 10% of FBS), where cells reverse their original phenotype within 72 h; original magnification: left panels, 10x; right panels, 20x. **b** Flow cytometry analysis after extracellular staining of TVM-A12 melanoma cells undergoing from adherent growth, in RPMI-1640 complete medium, to grape-like cellular aggregates in X-VIVO medium. **c** Flow cytometry analysis after intracellular staining of TVM-A12 melanoma cells undergoing from adherent growth, in RPMI-1640 complete medium, to grape-like cellular aggregates in X-VIVO medium. **d** Fluorescence microscopic analysis of Hoechst 33342 staining on living TVM-A12 cells growing in low serum X-VIVO medium (2% FBS); arrows point to putative cancer stem cells exhibiting low or negative Hoechst positivity in the nuclei. Original magnification: 40x. **e** Shows identification and characterization of the SP cells from TVM-A12 melanoma cells cultured for 72 h in RPMI-1640 complete medium or X-VIVO using Hoechst 33342 nucleic acid stain by FACS analysis. The FACS dot-plots displays the SP cells co-stained with CD133 marker preincubated with verapamil (*left panel*) and in the absence of verapamil (*middle panel*). Likewise, the FACS histograms plots (*right panel*) displays the percentage of CD133+ cells and the median of the relative fluorescence within the total SP cells in the absence of verapamil. **f** Relative mRNA expression of HERV-K env gene in TVM-A12 melanoma cells upon modification of culture conditions (** *p* < 0.001). Data represent the results of three independent experiments

In order to investigate the phenotype modifications of melanoma cells undergoing change of the microenvironment, the TVM-A12 melanoma cell line was cultured in a serum free stem cell medium (X-VIVO-15) and morphological features, cellular markers and antigens expression were characterized. As shown in Fig. 1a TVM-A12 cells, in the standard condition (RPMI + 10% FBS), displayed adherent phenotype and heterogeneous morphology, including small ovoid, spindle polygonal and large dendritic forms. However, when cultured in the X-VIVO medium, TVM-A12 cells showed a reduced proliferative potential, while the morphology shifted towards the formation of anchorage-independent grape-like cellular aggregates, characterized also by a dark colour due to melanin increase. In response to a gradual increment of serum (FBS) to X-VIVO medium, up to 10%, cells progressively reverted to an adherent phenotype within 72 h, with lower melanin content and morphology similar to the original one. These results demonstrate that TVM-A12 cells have an inherent characteristic of adaptive plasticity in response to the variations of the culture condition.

The phenotypic switching of TVM-A12 cells was characterized by flow cytometry analysis, using specific antibodies against selected cell surface markers of melanoma malignancy. The analysis was performed both on the adherent TVM-A12 cells, maintained in standard RPMI medium containing 10% FBS, and on the non-adherent grape-like cellular aggregates, induced by the X-VIVO medium (Fig. 1b, c). The morphological transition of TVM-A12 melanoma cells from adherent to suspended cellular aggregates, observed in X-VIVO medium, was accompanied by significant down-regulation of cell surface molecules such as HLA-I, Melan-A/MART-1 antigen, ICAM-1 and c-Kit, by maintenance or increase of tumour metastasis-associated markers such CXCR4 and CD10, and the increase of putative markers of CSCs such as CD133, Nestin, and NGF-R. For CD20 marker, which has been previously described as CSC marker in melanoma [3], not significant modification was determined. Moreover, flow cytometry analysis revealed that the addition of serum up to 10% to X-VIVO medium, other than re-establish the original morphology as already described in Fig. 1a, was also accompanied by the recovery of expression levels of the cellular markers of TVM-A12 grown in RPMI containing 10% FBS (data not shown).

Interestingly, the Hoechst 33342 extrusion test performed in living cells revealed also the presence in TVM-A12 of cells that exhibited similar property of CSCs, such enriched chemoresistance and tumorigenesis [3, 40]. X-VIVO medium with 2% of serum concentration was selected as condition in which non-adherent grape-like cellular aggregates and attached adherent cells were simultaneously present (Fig. 1d). The grapes in suspension showed (see arrows in Fig. 1d) different ability to efflux Hoechst. To better characterize this phenomenon, SP analysis was performed by flow cytometry (Fig. 1e). Using this method, the presence of a side population in TVM-A12 cell line was identified. Indeed, when the TVM-A12 cells cultured in standard RPMI medium were preincubated with verapamil before adding Hoechst 33342 dye, the percentage of SP cells significantly reduced, which is consistent with reports that verapamil blocks Hoechst efflux. Interestingly, part of these SP cells expressed CD133 marker, suggesting CD133 as one of the surface markers that potentially can be used to distinguish the population with stemness features in melanoma cells. Furthermore, in TVM-A12 cells cultured in X-VIVO, SP analysis identified a side population with a relative expression of the CD133 marker showing drug resistance features.

In light of our previous report that demonstrated the contribution of HERV-K to melanoma malignancy [26], we further investigated the activation of HERV-K in TVM-A12 melanoma cells during the expansion of CD133+ subpopulation in X-VIVO medium. To this end, using Real-time PCR, we evaluated the expression of HERV-K type II env gene (referred then in the text as HERV-K) in TVM-A12 cells upon their morphological transition from adherent to non-adherent grape-like cellular aggregates. Figure 1f shows a transcriptional activation of HERV-K concomitant to the phenotype-switching caused by exposure to X-VIVO medium. Interestingly, HERV-K transcriptional activity back to previous observed levels together with the reacquisition of the adherent phenotype upon re-exposure to RPMI with 10% FBS.

**Fig. 2:**
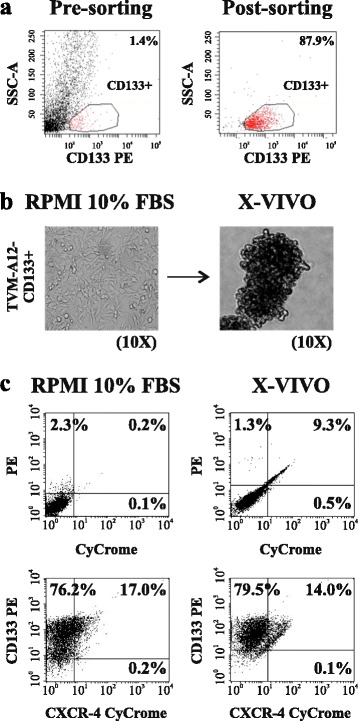
Cloned TVM-A12-CD133+ cells show adaptive plasticity similar to the parental TVM-A12 melanoma cells. **a** Sorting of CD133+ subpopulation from TVM-A12 cells by flow cytometry-based side population technique. **b** Phase contrast microscopy image of adherent cells (*left panel*) and grape-like cellular aggregates (*right panel*) of TVM-A12-CD133+ in RPMI-1640 and X-VIVO medium, respectively: magnification 10x. **c** Flow cytometry analysis of CD133 expression of adherent (*left panel*) and non-adherent (*right panel*) TVM-A12-CD133+ cells. Data represent the results of three independent experiments

The increased expression of markers related to both stem cells and malignancy during in X-VIVO culture and the SP analysis, suggested the expansion and maintenance of a stem cell-like CD133+ subpopulation. To further study this stem cell-like subpopulation, CD133^+^ cells were sorted through a flow cytometry based technique from the TVM-A12 (Fig. 2a). After sorting, cells were cloned by limiting dilution technique in standard RPMI medium with 10% FBS and stabilized as adherent cell lines with high CD133 expression level. In close analogy with the parental TVM-A12 cells, the exposure of TVM-A12-CD133+ cells to X-VIVO medium triggered the morphological transition from adherent to non-adherent phenotype (Fig. 2b) and maintained high levels of CD133 expression as shown by flow cytometry (Fig. 2c). Of note, the sorted TVM-A12-CD133+ cells can be maintained in the standard RPMI medium for a long time, showing gradually a reduction of CD133 expression. However, in the present study, we used only short cultured sorted cells analysed by FACS for CD133 expression immediately before the experiments, for the confirmation of the relative percentage of expression (in all the experiment >70%).

Taken together, these results demonstrated that TVM-A12 human melanoma cells have a change-prone phenotype responding to microenvironment modification, characterized by the expansion of a CD133+ melanoma subpopulation, enhanced expression of HERV-K and of markers related to immune evasion and metastasis formation.

### TVM-A12-CD133^+^ melanoma cells displayed enhanced self-renewing potential, migration and invasion capacity

**Fig. 3:**
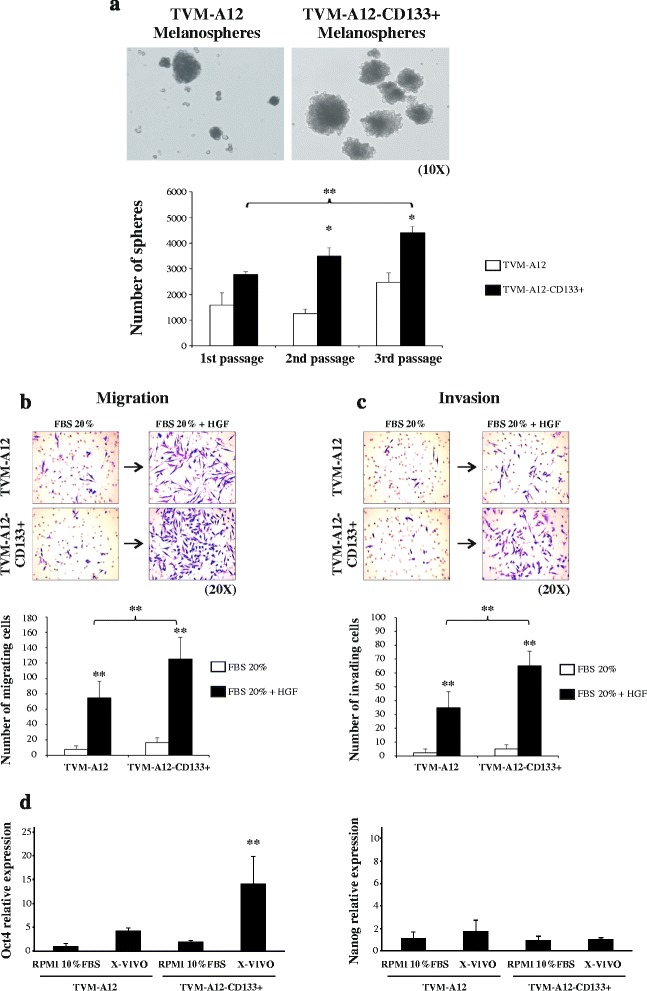
TVM-A12-CD133+ cells are endowed with CSCs features more than the parental TVM-A12 cells. **a** TVM-A12-CD133+ melanoma cells show higher self-renewing potential than TVM-A12 cells. *Top panels* represent the microscopy pictures of melanospheres from TVM-A12 cells (left) and TVM-A12-CD133+ cells (right); magnification 10x. Bar graph displays the self-renewing efficiency difference between TVM-A12 and TVM-A12-CD133+ cells in 2nd passage (*p* = 0.004) and 3rd passage (*p* = 0.012) and between 1st and 3rd passage of TVM-A12-CD133+ cells (** *p* < 0.001). **b** TVM-A12-CD133+ cells display higher migratory capacity than TVM-A12 cells. Top panels represent the microscopy pictures of TVM-A12 and TVM-A12-CD133+ migrated through the transwell insert: magnification 20x. Bar graph shows the migration capacity of TVM-A12 and TVM-A12-CD133+ cell lines in the presence or not of 40 ng/ml HGF (** *p* < 0.001). **c** TVM-A12-CD133+ cells display higher invasive capacity than TVM-A12 cells. Top panels represent the microscopy pictures of TVM-A12 and TVM-A12-CD133+ invasive cells through Matrigel coated transwell inserts: magnification 20x. Bar graph shows the invasive capacity difference between TVM-A12 and TVM-A12-CD133+ cell lines in the presence or not of 40 ng/ml HGF (** *p* < 0.001). ImageJ software was used to count total migrated and invasive cells. *p*-values (* *p* ≤ 0.050; ** *p* < 0.001). **d** Expression of the core stem cells transcriptional factor Oct4 (*left panel*) and Nanog (*right panel*). Data represent the results of three independent experiments

To verify if the TVM-A12 and TVM-A12-CD133+ cell lines exhibit some feature typical of cancer stem cells, we adopted sphere-forming assay, a technique widely used to characterize the CSCs self-renewing properties. Figure 3a shows that both cell lines were able to generate melanoma spheroids (melanospheres). Throughout the subsequent passages, the melanospheres forming efficiency of TVM-A12-CD133^+^ cells was significantly higher than the parental TVM-A12 cells (second passage *p* = 0.004, third passage *p* = 0.012), and only TVM-A12-CD133^+^cells were able to achieve a highly significant increment in the number of melanospheres between the first and third passage (*p* < 0.001). Together, these results show that TVM-A12-CD133^+^ cells have a greater self-renewing potential than the parental TVM-A12 cells.

We then compared the migratory/invasive capacity of TVM-A12 and TVM-A12-CD133+ cells using a transwell migration chamber in which cells were cultured for 48 h in RPMI with 20% FBS in the presence or not of 40 ng/ml hepatocyte growth factor (HGF). No significant difference in migration was found between the two cell lines cultured in RPMI medium with 20% FBS. However, the migration potential of both cell lines were strongly enhanced in the presence of 40 ng/ml HGF compared to the 20% FBS alone (*p* < 0.001), underlining the need of factors, typically present in tumour microenvironment known to promote migration of melanoma cells. Noteworthy, in the presence of HGF, the TVM-A12-CD133+ cells displayed a significantly higher cell migration potential than the parental TVM-A12 cells cultured in the same condition (*p* < 0.001) (Fig. 3b). Likewise, in a Matrigel coated inserts of transwell migration chambers, resembling the in vivo extracellular complex microenvironment, the presence of HGF stimulated invasion in both cell lines (*p* < 0.001), with the TVM-A12-CD133+ cells showing a significantly higher capacity to invade through the Matrigel compared to TVM-A12 cells (*p* < 0.001) (Fig. 3c).

Likewise, Real-time analysis of the core pluripotent regulatory transcription factors Oct4 and Nanog expression, which are well known to contribute the stemness and aggressive features of CSCs, revelead that both the parental TVM-A12 cell line and the sorted TVM-A12-CD133+ cells express these embryonic genes (Fig. 3d). Interestingly, the exposure to X-VIVO induced a statistically significant modulation of Oct4 in TVM-A12-CD133+ cells (*p* < 0.001). These results add further evidence of stemness features in the sorted CD133+ melanoma cells. These results confirm that TVM-A12-CD133+ cells have the inherent nature of stem cells properties that demarcates the defining feaures of CSCs.

On the whole, these results indicated that the parental TVM-A12 melanoma cells contain a CD133^+^ subpopulation endowed with features typical of CSCs such as high self-renewal and migratory/invasive capacity and embryonic genes expression.

### Concomitant CD133+ cells expansion and HERV-K activation in different melanoma cell lines

**Fig. 4:**
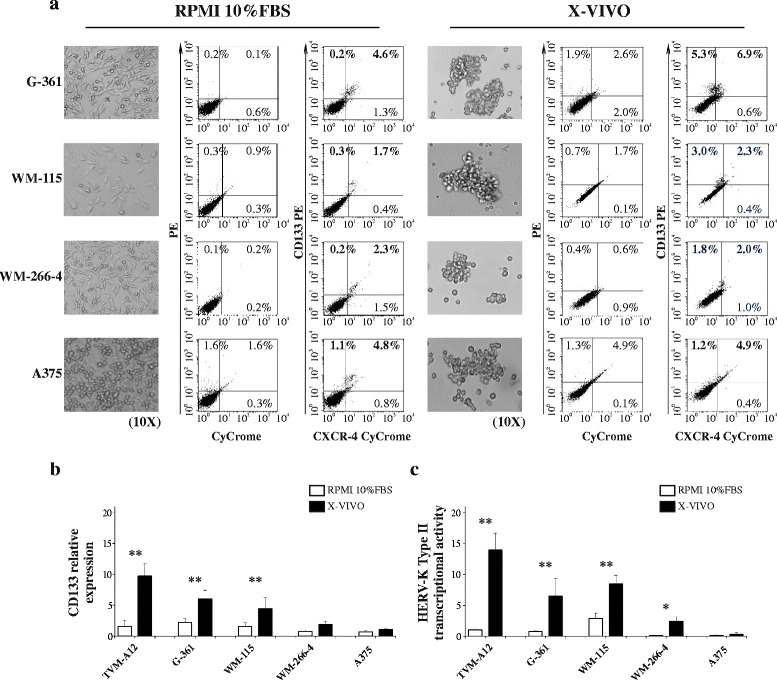
Different melanoma cell lines increase CD133 marker upon activation of HERV-K. **a** Morphological transition (*left panel*) and CD133 expression by flow cytometry (*right panel*) of human melanoma cell lines (G-361, WM-155, WM-266-4 and A375) upon exposure to X-VIVO medium: magnification 10x. Relative mRNA expression of CD133 (**b**) and HERV-K (**c**) in different melanoma cell lines upon modification of culture conditions. *p*-values (* *p* ≤ 0.050; ** *p* < 0.001). Data represent the results of three independent experiments

We then assessed if different melanoma cell lines, other than TVM-A12, were able to undergo morphological modification and express CD133 marker at higher level when cultivated in X-VIVO medium. Similar to TVM-A12, all the four melanoma cell lines (G-361, WM-155, WM-266-4 and A375) were able to undergo a morphological transition upon exposure to X-VIVO medium, but showed variable tendency to maintain and expand CD133+ subpopulation (Fig. 4a). Indeed, similar to TVM-A12, also G-361 and WM-115 cell lines showed an increased expression of CD133 when cultivated in X-VIVO (quadrant 1 + quadrant 2 of the dot-plots). On the other hand, WM-266-4 and especially A375 failed to increase CD133 marker in this culture condition. The different increased of CD133 expression among each cell lines was confirmed by Real time analysis (Fig. 4b). Intriguingly, this result is associated with the relative expression level of HERV-K (Fig. 1c). Indeed, the cell lines that failed to expand CD133 subpopulation in X-VIVO were those with lower or not significant HERV-K activation during exposure to microenvironment alterations.

These results highlight an association between the HERV-K activation under microenvironment alterations and the expansion of a CD133+ subpopulation with stemness features.

### Downregulation of HERV-K expression abolished the expansion of CD133+ melanoma subpopulation

**Fig. 5:**
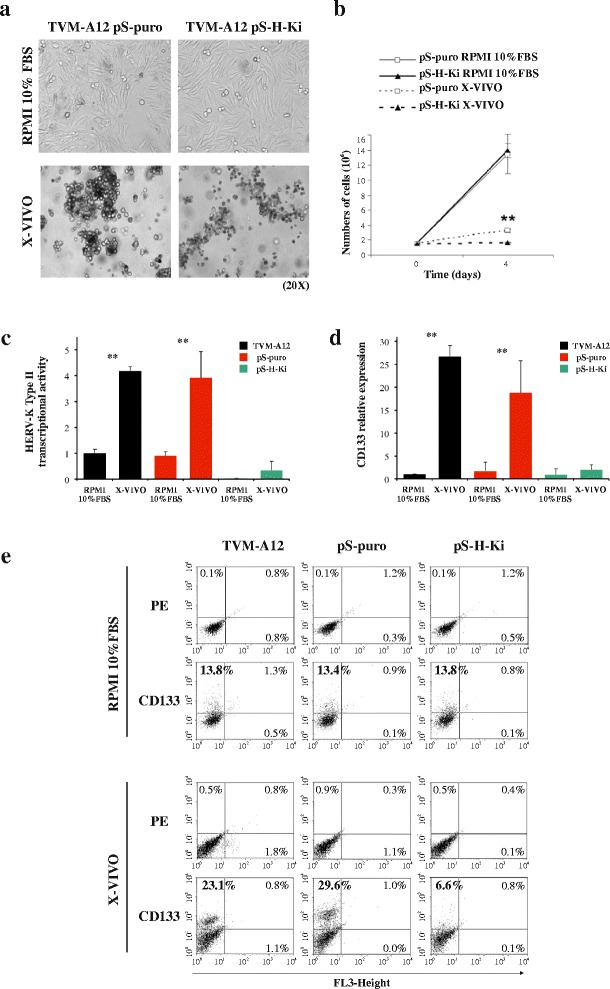
Downregulating of HERV-K expression by RNAi abolishes the expansion of CD133+ subpopulation. **a** Phase contrast microscopy image of adherent cells in RPMI-1640 medium (*upper panels*) and grape-like cellular aggregates in X-VIVO medium (*lower panels*) from TVM-A12pS-puro (non-interfered) and TVM-A12pS-H-Ki (interfered) cells: magnification 20x. **b** Cell counts. Relative mRNA expression of HERV-K env gene (**c**) and CD133 (**d**) in TVM-A12pS-puro and TVM-A12pS-H-Ki cells upon modification of culture conditions. **e** Flow cytometry analysis of CD133 expression shows HERV-K RNAi abolished the expansion and maintenance of CD133+ subpopulation in X-VIVO medium. Data represent the results of three independent experiments

We then investigated the role of HERV-K on cell plasticity and the maintenance of CD133^+^ cells in stem cell medium by downregulating HERV-K expression via RNA interference. TVM-A12 cells, infected with a retroviral vector expressing HERV-K-interfering shRNAs (pS-H-Ki construct) or with non-interfering retroviral vector alone (pS-puro construct), when cultured in the standard RPMI medium with 10% FBS, both exhibited an adherent phenotype. When cultured in X-VIVO medium, the pS-puro-infected cells switched towards the non-adherent aggregates formation, as the non-infected TVM-A12 cells, while pS-H-Ki-interfered cells were abnormal in X-VIVO showing less regular cellular aggregates morphology and reduction of cell growth compared to the TVM-A12pS-puro (Fig. 5a, b). Real-time PCR demonstrated that both TVM-A12 and TVM-A12pS-puro cell lines expressed higher levels of HERV-K env gene when cultivated in X-VIVO medium, compared to standard culture condition in RPMI medium with 10% FBS (*p* < 0.001). In contrast, as a result of HERV-K interference, in TVM-A12pS-H-Ki cells the expression of HERV-K env gene was strongly inhibited in both culture conditions (Fig. 5c).

In light of these results, we further investigated if the maintenance of the CD133+ cells in X-VIVO medium was dependent from the expression of HERV-K. Real-time PCR showed that in TVM-A12pS-puro, cultured in X-VIVO, the expression of CD133 mRNA was activated (*p* < 0.001), while in TVM-A12pS-H-Ki interfered cells the expression of CD133 was strongly inhibited (Fig. 5d). Flow cytometry analysis, also confirmed HERV-K dependency of the CD133+ subpopulation in X-VIVO. Indeed, the cell lines (TVM-A12, TVM-A12pS-puro or TVM-A12pS-H-Ki) cultured in standard RPMI medium with 10% FBS retained a similar percentage of CD133+ cells. However, when these cells were cultured in X-VIVO medium, both TVM-A12 and TVM-A12pS-puro cell lines were able to expand and maintain the subpopulation of CD133+ cells, while in the TVM-A12pS-H-Ki cell line the expansion was significantly abolished reaching a relative percentage of cell expressing CD133+ lower than in standard condition (Fig. 5e).

Throughout these results indicate that the downregulation of HERV-K prevents both the morphological transition of TVM-A12 cells towards a more malignant phenotype and the expansion of the CD133+ cells induced by X-VIVO medium.

### High susceptibility of TVM-A12-CD133+ melanoma cells to non-nucleoside reverse transcriptase inhibitors treatment

**Fig. 6:**
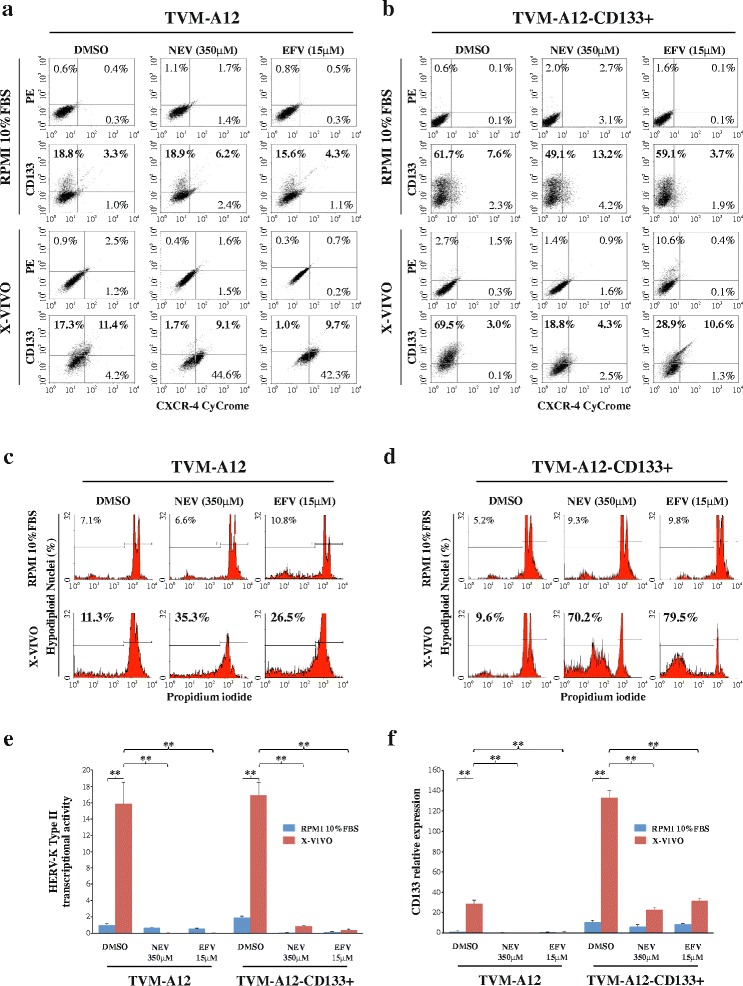
NNRTIs are effective to restrain HERV-K activation and induce apoptosis in TVM-A12 CD133+ cells. Effects of NNRTIs on the expansion and maintenance of CD133+ cells in TVM-A12 (**a**) and TVM-A12-CD133+ (**b**) cell lines analyzed by flow cytometry analysis. Effects of NNRTIs on apoptosis levels in TVM-A12 (**c**) and TVM-A12-CD133+ (**d**) analyzed by flow cytometry analysis after nuclei staining with propidium iodide. Relative mRNA expression of HERV-K env gene (**e**) and CD133 (**f**) analyzed by Real-time PCR. Data represent the results of three independent experiments

We have previously described that the non-nucleoside reverse transcriptase inhibitors (NNRTIs) Nevirapine and Efavirenz, currently used in the treatment of HIV patients, reduce proliferation and induce cell differentiation in TVM-A12 cells [41]. Thus, we wanted to verify the effect of NNRTIs treatment on the TVM-A12-CD133+ subpopulation. The data obtained showed that in X-VIVO medium, where HERV-K is highly activated and CD133+ cells expanded, the percentage of CD133 expressing cells within TVM-A12 cell line were significantly reduced from 28.7% in the control, to 10.8 and 10.7% upon treatment with nevirapine and efavirenz, respectively (Fig. 6a). Under the same conditions, the percentage of CD133^+^ expressing cells in TVM-A12-CD133+ cell line was also significantly reduced from 72.5% in control to 23.1 and 39.5%, after treatment with nevirapine and efavirenz, respectively (Fig. 6b). Moreover, in X-VIVO medium apoptosis was moderately induced in TVM-A12 cell line treated with nevirapine (35.3%) and efavirenz (26.5%), as compared to the 11.3% in control sample (Fig. 6c), while a significantly higher level of apoptosis was induced in TVM-A12-CD133+ cell line by nevirapine (70.2%) and efavirenz (79.5%), compared to the 9.6% in control sample (Fig. 6d). The analysis by Real-time PCR, showed that the effect of the inhibitors efficiently prevented the activation of HERV-K in TVM-A12 cells and TVM-A12-CD133+ cell lines (*p* < 0.001) (Fig. 6e). Interestingly, in both the cell lines, the down-regulation of HERV-K was accompanied by a parallel down-regulation of CD133 expression (*p* < 0.001) (Fig. 6f). Thus, these results demonstrated that inhibition of endogenous reverse transcriptase by the NNRTIs prevents the activation of HERV-K and is concomitantly accompanied by the CD133 down-regulation and the inability to expand and maintain the CD133+ cells.

## Discussion

In different types of tumors CSCs have been shown to possess an inherent characteristic of cellular plasticity to resist and survive in harsh microenvironment conditions, encountered during tumor progression [2]. In melanoma, CSCs plasticity has been related to intratumoral heterogeneity, metastasis formation, resistance to chemotherapy and tumor recurrence [7].

In the present study TVM-A12 human melanoma cell line, originated from a primary lesion, showed peculiar plasticity features upon the modification of microenvironment by cultivation in a stem cell medium, switching from an adherent phenotype towards an anchorage-independent grape-like cellular aggregates formation. This morphological transition was accompanied by the acquisition of more aggressive and invasive characteristics such as significantly reduced expression of cell surface markers HLA-I, MelanA/MART-1 and ICAM-1, all involved in the host immune response [42–44]. This is in agreement with our previous paper, in which we described the contribution of HERV-K in reducing the immunogenicity of melanoma cells [26] and therefore favoring the evasion of the tumour from the host immune responses [1]. In addition, herein increased expression of stem cells and metastasis related markers CD133, nestin, CXCR4, and NGF-R along with continued high-level of CD10 expression were observed, which usually delineates progression to advanced stage of melanoma and metastatic tumour formation [4, 45]. Moreover, the survival of TVM-A12 cells in non-adherent condition implied these cells may have developed a mechanism to resist and thereby survive after detachment. Likewise, metastatic cancer cells are able to survive after detachment from their primary site by developing resistance to a specialized form of cell death called anoikis [46, 47].

It was described that HERV-K expression may support cancer cells to go through fast gene plasticity to adopt new microenviromental changes [16, 48]. Hence, the phenotype-switching of TVM-A12 cells, occurring in the stem cell medium, and the concomitant HERV-K activation, both reversible after serum addition, highlight that TVM-A12 cells harbor a high capacity to adapt to the microenvironment modifications, while maintaining an inherent ductility to reestablish the original features. Intriguingly, silencing HERV-K in TVM-A12 cells attenuated their plasticity towards invasive malignant phenotype, which seems to affirm that in melanoma cells the aberrant activation of HERV-K is associated with the process of invasive phenotype-switching, occurring in response to microenvironmental modification. This finding corroborate previous reports demonstrating melanoma cells undergo the process of phenotype-switching to promote metastatic state while maintaining the stem cell-like identity [14, 49].

In this study the expansion of the CD133+ subpopulation and the concomitant up-regulation of HERV-K were observed during the morphological transition of TVM-A12 cells towards the non-adherent malignant phenotype. Interestingly, we found that these phenomena are not limited to TVM-A12 melanoma cell line, rather they are seemingly common traits shown with different extent by other type of commercially available melanoma cell lines. Indeed, all the cell lines tested showed various basal expressions of CD133 marker and aptitude to expand this subpopulation, concomitantly with HERV-K activation, when maintained in stem cell medium. Remarkably, blocking of HERV-K expression through siRNA significantly abolished the expansion and maintenance of CD133+ subpopulation within TVM-A12 cells in the stem cell medium. It is also well documented that the main defining characteristics of putative CSCs are self-renewing and metastatic potential [50] as well as the expression of pluripotent stem cells transcription factors, such as Nanog, Oct4, and Sox2 [51]. From this perspective, this study showed that the TVM-A12-CD133+ cells, which are dependent on HERV-K activation, are endowed with the defining characteristics of CSCs as evidenced by their enhanced self-renewal ability, higher migratory and invasive capacities and expression of the stem cell markers Oct4 and Nanog. This study also showed CD133+ subpopulation is partly present in the Hoechst dye-effluxing SP cells, which possess many CSCs properties [40]. These findings seems to indicate that HERV-K is also involved in the stemness genomic networks of the CSCs model in melanoma tumor. In support to this hypothesis, compelling evidences suggest that expression of HERVs have a direct participatory role for the maintenance of human embryonic stem cells (hESCs) and induced pluripotent stem cells (iPSCs), and, their activation or re-activation have been evinced as a marker of pluripotency [16, 31]. Likewise, HERV-K has been associated with cellular transformation and carcinogenesis [16, 23, 25]. Of note, very recently has been demonstrated the essential role of HERV-K env protein activation for tumorigenesis and metastasis of breast cancer cells [52]. In this regard, our study presents the first evidence that demonstrate the fundamental role of HERV-K in the expansion and maintenance of CSCs in melanoma tumor during phenotype switching under microenvironment modifications.

It has been shown that microenvironmental stress conditions influence the expression of HERVs in cancer cells through epigenetic modification, most likely through DNA hypomethylation of relevant retroviral genes [23]. Moreover, it was reported that DNA hypomethylation is accounted as an important determining factor of CD133 expression in ovarian and glioma cancer cells [53, 54]. Global DNA hypomethylation is a hallmark of many cancers [55] and HERV-K (HML-2) hypomethylation in particular has been reported in melanoma cell lines [56]. Hence, the aberrant activation of HERV-K in melanoma cells could depend on the epigenetic modification induced by change in tumor microenvironment that affects the DNA methylation state. Taken together, this study revealed that the expansion of CD133+ melanoma cells with stemness features are dependent on HERV-K activation during microenvironment modifications, likely mediated by stress condition.

We previously reported that inhibition of retroelements-encoded RT by NNRTIs or by RNA interference, resulted in anti-proliferative, pro-differentiating effects in melanoma cells and reduction of melanoma tumor growth in mice models [27, 41, 57]. Interestingly, here we found that efavirenz and nevirapine were able to halt the expansion and maintenance of CD133+ melanoma cells inducing high levels of apoptosis and were effective to restrain the activation of HERV-K during microenvironmental modification. Therefore, the high sensitivity of TVM-A12-CD133+ cells to NNRTIs suggests a specific requirement of HERV-K expression to sustain this subpopulation in melanoma cells during microenvironmental modifications. This result confirms our previous findings that retroelements are key players in melanoma tumor progression [41, 57–59], demonstrating for the first time the specific role of HERV-K in the generation and survival of CD133+ melanoma CSCs. These findings open new perspectives for targeting the putative CSCs that express high-level of HERV-K and for developing new possible therapeutic strategies based on tumor cells differentiation.

## Conclusions

Taken overall, findings from this study demonstrated the involvement of HERV-K in the plasticity of human melanoma cells undergoing change of microenvironment, with a peculiar role on the CD133+ subpopulation endowed with CSC features. In light of this, and based on previous evidences reported by our group and others, it possible to address HERV-K as one of the important actors in the dynamics of melanoma development. Particularly, we demonstrated that HERV-K accompany phenotype switching and is strictly required to sustain the more malignant CD133+ subpopulation of cancer cells with stemness features.

## References

[1] Frank NY, Schatton T, Frank MH (2010). The therapeutic promise of the cancer stem cell concept. J Clin Invest.

[2] Pattabiraman DR, Weinberg RA (2014). Tackling the cancer stem cells-what challenges do they pose?. Nat Rev Drug Discov.

[3] Shakhova O, Sommer L (2013). Testing the cancer stem cell hypothesis in melanoma: the clinics will tell. Cancer Lett.

[4] Zimmerer RM, Korn P, Demougin P, Kampmann A, Kokemüller H, Eckardt AM (2013). Functional features of cancer stem cells in melanoma cell lines. Cancer Cell Int.

[5] Yu X, Lin Y, Yan X, Tian Q, Li L, Lin EH (2011). CD133, Stem Cells, and Cancer Stem Cells: Myth or Reality?. Curr Colorectal Cancer Rep.

[6] Monzani E, Facchetti F, Galmozzi E, Corsini E, Benetti A, Cavazzin C (2007). Melanoma contains CD133 and ABCG2 positive cells with enhanced tumourigenic potential. Eur J Cancer.

[7] Girouard SD, Murphy GF (2011). Melanoma stem cells: not rare, but well done. Lab Invest.

[8] Lai CY, Schwartz BE, Hsu MY (2012). CD133+ melanoma subpopulations contribute to perivascular niche morphogenesis and tumorigenicity through vasculogenic mimicry. Cancer Res.

[9] Adini A, Adini I, Ghosh K, Benny O, Pravda E, Hu R (2013). The stem cell marker prominin-1/CD133 interacts with vascular endothelial growth factor and potentiates its action. Angiogenesis.

[10] El-Khattouti A, Selimovic D, Haïkel Y, Megahed M, Gomez CR, Hassan M (2014). Identification and analysis of CD133(+) melanoma stem-like cells conferring resistance to taxol: An insight into the mechanisms of their resistance and response. Cancer Lett.

[11] Rappa G, Fodstad O, Lorico A (2008). The Stem Cell‐Associated Antigen CD133 (Prominin‐1) Is a Molecular Therapeutic Target for Metastatic Melanoma. Stem Cells.

[12] AbdusSamad M, Gaur A, Zhou H, Zapas JL, Simbulan-Rosenthal CM, McCarron EC (2015). CD133 knockdown sensitizes melanoma to kinase inhibitors. Cancer Res.

[13] Van den Hurk K, Niessen H, Veeck J, Van den Oord JJ, Van Steensel M, Zur Hausen A (2012). Genetics and epigenetics of cutaneous malignant melanoma: a concert out of tune. Biochim Biophys Acta.

[14] Hook KS, Goding CR (2010). Cancer stem cells versus phenotype‐switching in melanoma. Pigment Cell Melanoma Res.

[15] Magiorkinis G, Belshaw R, Katzourakis A (2013). ‘There and back again’: revisiting the pathophysiological roles of human endogenous retroviruses in the post-genomic era. Philos Trans R Soc Lond B Biol Sci.

[16] Göke J, Ng HH (2016). CTRL+ INSERT: retrotransposons and their contribution to regulation and innovation of the transcriptome. EMBO Rep.

[17] Robbez-Masson L, Rowe HM (2015). Retrotransposons shape species-specific embryonic stem cell gene expression. Retrovirology.

[18] Okahara G, Matsubara S, Oda T, Sugimoto J, Jinno Y, Kanaya F (2004). Expression analyses of human endogenous retroviruses (HERVs): tissue-specific and developmental stage-dependent expression of HERVs. Genomics.

[19] Balestrieri E, Pica F, Matteucci C, Zenobi R, Sorrentino R, Argaw-Denboba A (2015). Transcriptional Activity of Human Endogenous Retroviruses in Human Peripheral Blood Mononuclear Cells. Biomed Res Int.

[20] Balestrieri E, Arpino C, Matteucci C, Sorrentino R, Pica F, Alessandrelli R (2012). HERVs expression in autism spectrum disorders. PLoS One.

[21] Balestrieri E, Pitzianti M, Matteucci C, D’Agati E, Sorrentino R, Baratta A (2014). Human endogenous retroviruses and ADHD. World J Biol Psychiatry.

[22] Young GR, Stoye JP, Kassiotis G (2013). Are human endogenous retroviruses pathogenic? An approach to testing the hypothesis. Bioessays.

[23] Cegolon L, Salata C, Weiderpass E, Vineis P, Palù G, Mastrangelo G (2013). Human endogenous retroviruses and cancer prevention: evidence and prospects. BMC Cancer.

[24] Downey RF, Sullivan FJ, Wang-Johanning F, Ambs S, Giles FJ, Glynn SA (2015). Human endogenous retrovirus K and cancer: Innocent bystander or tumorigenic accomplice?. Int J Cancer.

[25] Hohn O, Hanke K, Bannert N (2013). HERV-K(HML-2), the best preserved family of HERVs: endogenization, expression, and implications in health and disease. Front Oncol.

[26] Serafino A, Balestrieri E, Pierimarchi P, Matteucci C, Moroni G, Oricchio E (2009). The activation of human endogenous retrovirus K(HERV-K) is implicated in melanoma cell malignant transformation. Exp Cell Res.

[27] Oricchio E, Sciamanna I, Beraldi R, Tolstonog GV, Schumann GG, Spadafora C (2007). Distinct roles for LINE-1 and HERV-K retroelements in cell proliferation, differentiation and tumor progression. Oncogene.

[28] Schmitt K, Reichrath J, Roesch A, Meese E, Mayer J (2013). Transcriptional profiling of human endogenous retrovirus group HERV-K (HML-2) loci in melanoma. Genome Biol Evol.

[29] Reiche J, Pauli G, Ellerbrok H (2010). Differential expression of human endogenous retrovirus K transcripts in primary human melanocytes and melanoma cell lines after UV irradiation. Melanoma Res.

[30] Armbruester V, Sauter M, Roemer K, Best B, Hahn S, Nty A (2004). Np9 protein of human endogenous retrovirus K interacts with ligand of numb protein X. J Virol.

[31] Fuchs NV, Loewer S, Daley GQ, Izsvák Z, Lower J, Lower R (2013). Human endogenous retrovirus K (HML-2) RNA and protein expression is a marker for human embryonic and induced pluripotent stem cells. Retrovirology.

[32] Krone B, Kölmel KF, Henz BM, Grange JM (2005). Protection against melanoma by vaccination with bacilleCalmette–Guérin (BCG) and/or vaccinia: an epidemiology-based hypothesis on the nature of a melanoma risk factor and its immunological control. Eur J Cancer.

[33] Mangeney M, Pothlichet J, Renard M, Ducos B, Heidmann T (2005). Endogenous retrovirus expression is required for murine melanoma tumor growth in vivo. Cancer Res.

[34] Dong J, Huang G, Imtiaz R, Xu F. The Potential Importance of K Type Human Endogenous Retroviral Elements in Melanoma Biology. INTECH Open Access Publisher 2013; DOI: 10.5772/55264. Article. Google Scholar

[35] Huang G, Li Z, Wan X, Wang Y, Dong J (2013). Human endogenous retroviral K element encodes fusogenic activity in melanoma cells. J Carcinog.

[36] Wang AX, Qi XY (2013). Targeting RAS/RAF/MEK/ERK signaling in metastatic melanoma. IUBMB Life.

[37] Melino G, Sinibaldi-Vallebona P, D’Altri S, Annichiarico-Petruzzelli M, Rasi G, Catani MV (1993). Characterization of three melanoma cell lines (TVM-A12, TVM-A197, TVM-BO) sensitivity to lysis and effect of retinoic acid. Clin Chem Enzymol Commun.

[38] Hirschmann-Jax C, Foster AE, Wulf GG, Nuchtern JG, Jax TW, Gobel U (2004). A distinct “side population” of cells with high drug efflux capacity in human tumor cells. Proc Natl Acad Sci U S A.

[39] Goodell MA, Brose K, Paradis G, Conner AS, Mulligan RC (1996). Isolation and functional properties of murine hematopoietic stem cells that are replicating in vivo. J Exp Med.

[40] Wouters J, Stas M, Gremeaux L, Govaere O, Maes H, Agostinis P (2013). The human melanoma side population displays molecular and functional characteristics of enriched chemoresistance and tumorigenesis. PLoS One.

[41] Sciamanna I, Landriscina M, Pittoggi C, Quirino M, Mearelli C, Beraldi R (2005). Inhibition of endogenous reverse transcriptase antagonizes human tumor growth. Oncogene.

[42] Kurnick JT, Ramirez-Montagut T, Boyle LA, Andrews DM, Pandolfi F, Durda PJ (2001). A novel autocrine pathway of tumor escape from immune recognition: melanoma cell lines produce a soluble protein that diminishes expression of the gene encoding the melanocyte lineage melan-A/MART-1 antigen through down-modulation of its promoter. J Immunol.

[43] Mendez R, Aptsiauri N, Del Campo A, Maleno I, Cabrera T, Ruiz-Cabello F (2009). HLA and melanoma: multiple alterations in HLA class I and II expression in human melanoma cell lines from ESTDAB cell bank. Cancer Immunol Immunother.

[44] Hamaï A, Meslin F, Benlalam H, Jalil A, Mehrpour M, Faure F (2008). ICAM-1 has a critical role in the regulation of metastatic melanoma tumor susceptibility to CTL lysis by interfering with PI3K/AKT pathway. Cancer Res.

[45] Oba J, Nakahara T, Hashimoto-Hachiya A, Liu M, Abe T, Hagihara A (2016). CD10-Equipped Melanoma Cells Acquire Highly Potent Tumorigenic Activity: A Plausible Explanation of Their Significance for a Poor Prognosis. PLoS One.

[46] Liu W, Vivian CJ, Brinker AE, Hampton KR, Lianidou E, Welch DR (2014). Microenvironmental influences on metastasis suppressor expression and function during a metastatic cell’s journey. Cancer Microenviron.

[47] Fofaria NM, Srivastava SK (2014). Critical role of STAT3 in melanoma metastasis through anoikis resistance. Oncotarget.

[48] Löwer R, Löwer J, Kurth R (1996). The viruses in all of us: characteristics and biological significance of human endogenous retrovirus sequences. Proc Natl Acad Sci U S A.

[49] Li FZ, Dhillon AS, Anderson RL, McArthur G, Ferrao PT (2015). Phenotype switching in melanoma: implications for progression and therapy. Front Oncol.

[50] Liao WT, Ye YP, Deng YJ, Bian XW, Ding YQ (2014). Metastatic cancer stem cells: from the concept to therapeutics. Am J Stem Cells.

[51] Liu A, Yu X, Liu S (2013). Pluripotency transcription factors and cancer stem cells: small genes make a big difference. Chin J Cancer.

[52] Zhou F, Li M, Wei Y, Lin K, Lu Y, Shen J, Johanning GL, Wang-Johanning F. Activation of HERV-K Env protein is essential for tumorigenesis and metastasis of breast cancer cells. Oncotarget. 2016 [Epub ahead of print] Aug 20. PubMed. Google Scholar 10.18632/oncotarget.11455 PMC5356647 27557521

[53] Baba T, Convery PA, Matsumura N, Whitaker RS, Kondoh E, Perry T (2009). Epigenetic regulation of CD133 and tumorigenicity of CD133+ ovarian cancer cells. Oncogene.

[54] Wu X, Wu F, Xu D, Zhang T (2016). Prognostic significance of stem cell marker CD133 determined by promoter methylation but not by immunohistochemical expression in malignant gliomas. J Neurooncol.

[55] Ehrlich M (2009). DNA hypomethylation in cancer cells. Epigenomics.

[56] Stengel S, Fiebig U, Kurth R, Denner J (2010). Regulation of human endogenous retrovirus‐K expression in melanomas by CpG methylation. Genes Chromosomes Cancer.

[57] Sinibaldi-Vallebona P, Matteucci C, Spadafora C (2011). Retrotransposon-encoded reverse transcriptase in the genesis, progression and cellular plasticity of human cancer. Cancers (Basel).

[58] Sciamanna I, Gualtieri A, Piazza PV, Spadafora C (2014). Regulatory roles of LINE-1-encoded reverse transcriptase in cancer onset and progression. Oncotarget.

[59] Sciamanna I, De Luca C, Spadafora C (2016). The Reverse Transcriptase Encoded by LINE-1 Retrotransposons in the Genesis, Progression, and Therapy of Cancer. Front Chem.

